# Non‐breeding social behaviour as an investment in reducing future territoriality costs

**DOI:** 10.1111/brv.70066

**Published:** 2025-08-11

**Authors:** Samuele Ramellini, Brendah Nyaguthii, Ettore Camerlenghi, Damien R. Farine

**Affiliations:** ^1^ Division of Ecology and Evolution Research School of Biology, Australian National University 46 Sullivans Creek Road, Acton Canberra Australia; ^2^ Collegium Helveticum, ETH Zurich/STW Schmelzbergstrasse 25 Zürich Switzerland; ^3^ Department of Evolutionary Biology and Environmental Studies University of Zurich 190 Winterhurerstrasse Zürich Switzerland; ^4^ Department of Collective Behaviour Max Planck Institute of Animal Behavior Am Obstberg 1 Radolfzell Germany

**Keywords:** territoriality, territory establishment, sociality, non‐breeding season, seasonal breeding, interdependence, dear‐enemy effect, territory neighbours, multilevel society, carry‐over effects

## Abstract

Territoriality is costly, and animals should adopt strategies to cope with these costs. Seasonal territoriality for breeding – a common strategy in many groups of animals – can reduce costs during the non‐breeding season but requires establishing new territories every breeding season. Many seasonal breeders also become more tolerant of conspecifics during the non‐breeding season and form social groups containing many individuals. Recent evidence has suggested that these social associations are not random and can entail carry‐over effects extending into the following breeding season. Here, we propose that one strategy that seasonal, territorial breeders may employ is to use non‐breeding social behaviour to reduce future breeding territoriality costs, through a dear‐enemy‐like effect. Specifically, by being social during the non‐breeding season with previous territorial neighbours, individuals can increase both their and their neighbour's survival, and jointly defend and exploit common territorial areas, ultimately reducing neighbourhood turnover. Reduced neighbourhood turnover can then facilitate re‐forming prior territorial boundaries, thereby offsetting the costs of territory establishment and facilitating earlier breeding (which can significantly increase reproductive output). We review evidence supporting our hypothesis and provide predictions and future research directions to bridge current gaps in understanding the link between non‐breeding social behaviours and breeding territoriality.

## I. INTRODUCTION

Many animals maintain a territory only during the breeding season and can range more broadly outside of this season – a strategy called seasonal territoriality. This strategy is widespread in the animal kingdom, including in birds (Tobias *et al*., 2016), mammals (Bronson, 2009), amphibians (Mathis *et al*., 1995) and fishes (Kramer, 1978). Shifts or expansion in space use can arise because of limitations due to resources (e.g. the territory area cannot support the individuals during harsher seasons; Schoener, 1983) or be facilitated because territorial behaviours impose significant costs on individuals that are not worth paying when conditions are unfavourable (Schoener, 1987; Gill & Wolf, 1975). Such costly behaviours used to maintain territories can range from song displays [e.g. in birds (Searcy & Beecher, 2009) or amphibians (Chuang, Kam & Bee, 2017)] and visual displays [e.g. fish (Frommen & Fox, 2019) or reptiles (Stamps, 1983)] to highly aggressive behaviours, such as physical contests (e.g. in social ants; Thomas *et al*., 2006). Individuals thus incur significant physiological costs (Oberweger & Goller, 2001; Marler *et al*., 1995) as well as an increased risk of predation (Schmidt & Belinsky, 2013) or injuries/death from territorial disputes (Watts *et al*., 2006; Thomas *et al*., 2006). While seasonal breeding can reduce the costs of defending territories year‐round, it also potentially introduces new costs, specifically those associated with (re‐)establishing territories at the start of each breeding season (Stamps, 1987; Stamps & Krishnan, 1997). Establishment costs, including the risk of missing out altogether on gaining a territory, could then select for strategies that can reduce the risk of paying these costs. Yet, to date there has been relatively little attention paid to potential links between non‐breeding behaviour and territorial behaviours in the lead up to, and during, the following breeding season.

A seasonal shift in territoriality can correspond to many behavioural changes at the individual level. At one extreme, we can observe a complete temporary migration (Newton, 2011) or permanent relocation (Teitelbaum & Mueller, 2019) of the breeding population to new, distant locations. At the other extreme, territories can break down in sedentary, resident species, meaning that individuals are largely free to move anywhere, which can lead to a temporary seasonal change of home ranges, a pattern especially common in birds (Camerlenghi *et al*., 2021; Griesser *et al*., 2009; Bell, 1985; Papageorgiou & Farine, 2021), but also, although less evidently, in some mammals [Csányi *et al*., 2024; but see Pettett *et al*. (2018) for no seasonal differences or Priotto, Steinmann & Polop (2002) for the opposite]. Such changes in home‐range size are generally assumed to be particularly important to find resources in harsh conditions (Teitelbaum & Mueller, 2019). Thus, the loss of territoriality during the non‐breeding season is likely to be linked to both avoiding the costs of maintaining territories and the need to access resources that are essential for survival.

One consequence of the reduced territoriality, allowing individuals to move over larger areas, is an increase in the chance of encountering conspecifics (Beck *et al*., 2023). For example, in superb fairywrens *Malurus cyaneus* and red‐backed fairywrens *M. melanocephalus*, as well as in apostlebirds *Struthidea cinerea*, the overlap among breeding groups (which maintain cohesion outside of breeding) is larger during the non‐breeding season than during the breeding season (Welklin *et al*., 2023; Camerlenghi *et al*., 2021; Griesser *et al*., 2009). Similar patterns have been observed in mammals, such as in captive brushtail possums *Trichosurus vulpecula* (Day *et al*., 2000), and fish, such as the longnose filefish *Oxymonacanthus longirostris* (Kokita & Nakazono, 1999), although they are likely much more common in birds and can even be reversed in some mammals (e.g. stoats *Mustela erminea* are strictly territorial during the non‐breeding season but overlap during the breeding season; Erlinge & Sandell, 1986). Recent studies focusing on social behaviour outside of the breeding season have also revealed that the resulting social associations during the non‐breeding season are far from being random encounters among individuals. For example, migratory golden‐crowned sparrows *Zonotrichia atricapilla* have consistent social communities at their wintering grounds that are maintained across years (Shizuka *et al*., 2014) and, despite forming large roosts with open membership, social associations among sulphur‐crested cockatoos *Cacatua galerita* are largely between kin (Penndorf *et al*., 2023). Being strategic in who to socialise with can then provide opportunities for individuals to reap important benefits across several components of life history.

It is now well established that individuals can accrue immediate benefits from being social, especially during the harsher conditions that they experience during the non‐breeding season. For example, more socially connected great tits *Parus major*, blue tits *Cyanistes caeruleus*, and marsh tits *Poecile palustris* are more likely to find novel food sources (Aplin *et al*., 2012), and social bonds are more important than foraging ties in predicting the acquisition of social information about food by European starlings *Sturnus vulgaris* (Boogert *et al*., 2014). While these benefits have been extensively quantified in birds, similar patterns have also been noted in social mammals (Qi *et al*., 2023) and reptiles (e.g. communal denning in rattlesnakes *Crotalus horridus*; Clark *et al*., 2012). Such benefits are expected to increase survival – and therefore increase the chance that the individual will be able to reproduce in the future – when they offset costs arising from, for example, disease risk (Ashby & Farine, 2022; Evans *et al*., 2020). However, while these studies suggest that being more social, in general, can be beneficial, it is now also becoming clear that who individuals are social with during non‐breeding may have consequences on their reproductive outcomes in the following breeding season.

There is now increasing awareness of individual carry‐over effects occurring between seasons (Marra *et al*., 2015; Harrison *et al*., 2011), and evidence is emerging that non‐breeding sociality can impact dynamics in subsequent breeding seasons. For example, breeding success in long‐tailed tits *Aegithalos caudatus* is substantially enhanced for nests that have helpers (Hatchwell, Gullett & Adams, 2014), and future helping behaviour is linked to non‐breeding associations (Napper & Hatchwell, 2016). In great tits, males are also more likely to breed in the spring if they disperse into the future breeding area earlier than their competitors do (Farine & Sheldon, 2015). There is also evidence that pair bonds (a male and female that eventually breed together) begin to form within non‐breeding social groups, long before the onset of breeding (Maldonado‐Chaparro, Forstmeier & Farine, 2021; Teitelbaum, Converse & Mueller, 2017; Culina, Firth & Hinde, 2020), and male and female blue tits that associate often during the non‐breeding season are also more likely to have extra‐pair offspring during the following breeding season (Beck, Farine & Kempenaers, 2020). Similarly, in primates (Feldblum *et al*., 2021) but also other mammals (Cameron, Setsaas & Linklater, 2009), sociality outside of a breeding context is hypothesised to play an important role in future reproductive success. Thus, social behaviours outside of breeding may reflect strategies that allow individuals to deal with some of the challenges (finding a mate, establishing a territory) that arise at the onset of the reproductive season.

**Fig. 1:**
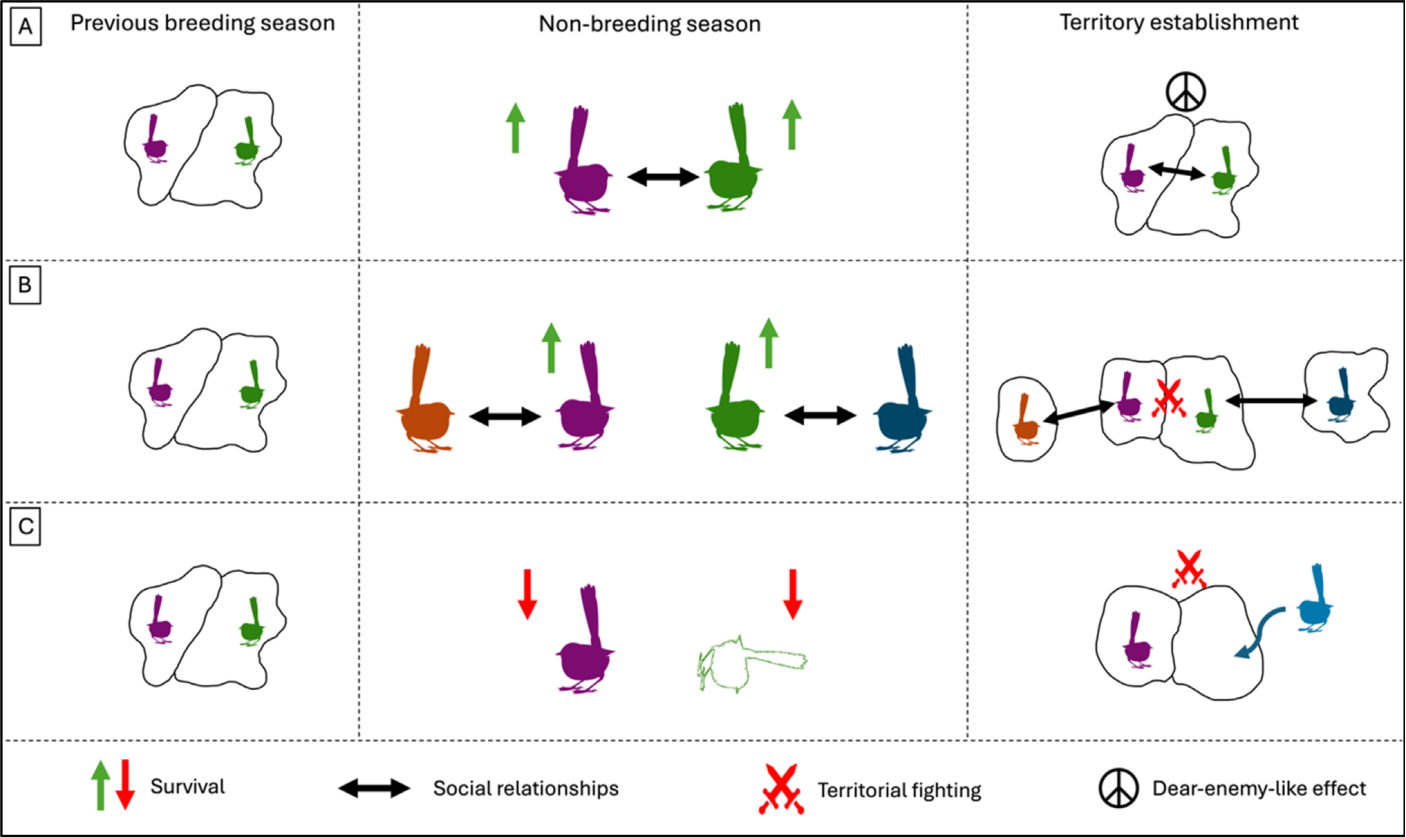
Non‐breeding social behaviour can reduce the costs of establishing territories among neighbours. At the end of the breeding season (left panels), individuals can strategically decide who to associate with during the non‐breeding season (central panels). Non‐breeding sociality has direct, short‐term benefits in terms of survival (upwards‐pointing green arrows), but is also likely to affect behaviours during the following breeding season (right panels). These latter effects are predicted to depend on who the individuals associated with. If individuals preferentially associated with their territory neighbours, costs of territory establishment could be reduced through a dear‐enemy‐like effect (A). On the contrary, if social relationships were independent of individuals being spatial neighbours, survival may still be favoured but the costs of future territory establishment are less likely to be offset (B). Finally, non‐social individuals could experience increased mortality, driving population turnover that would allow new individuals to enter the breeding population and require new territories to be established (C).

Here, we propose a novel hypothesis that in seasonal, territorial breeders, social behaviour during the non‐breeding season can act to reduce costs arising from territorial disputes during the subsequent breeding season (Fig. 1). Establishing territorial boundaries at the onset of the breeding season is costly both in terms of the energetic costs but also as an opportunity cost if it reduces investment in breeding activities (e.g. by delaying nesting) or increases the chance of losing the territory altogether (Ord, 2021). For instance, both New Holland honeyeaters *Phylidonyris novaehollandiae* and white‐cheeked honeyeaters *P. niger* have a significantly higher attack rate in the early phase rather than in the core phase of the breeding season (Armstrong, 1991). Similarly, male pumpkinseed sunfish *Lepomis gibbosus* show the strongest aggressive response during the early breeding season and progressively reduce attack rates throughout the breeding season (Colgan & Gross, 1977). Paying these costs can reduce body condition and result in a higher probability of losing a territory during the establishment phase (Mesterton‐Gibbons & Sherratt, 2016), ultimately preventing reproduction. Avoiding such costly actions would clearly be beneficial as it allows individuals to devote this energy towards breeding activities.

Our premise is that if breeding neighbourhoods can remain stable from one breeding season to the next, then individuals can more easily re‐establish previous territory boundaries through a process like the dear‐enemy effect (Section II). The dear‐enemy effect is a widespread phenomenon observed across several groups of animals with individuals reducing investment in territory defence against neighbours (or more familiar individuals), because these pose a lower threat than strangers. Such a process could likely also occur across seasons, whereby two territory owners that were previously neighbours could re‐establish their prior territory boundaries with minimal conflict (Section III). If this is the case, then the benefits reaped from reduced costs of territory establishment would promote strategies that increase the likelihood of neighbours surviving or remaining in the area. One such strategy is being social: sociality among neighbours during the non‐breeding season could allow individuals to increase their neighbours' survival (through group‐size effects) and allow for communal defence of joint territory areas, both of which would result in a lower neighbourhood turnover.

When neighbourhoods remain stable and individuals maintain residency over multiple seasons, there are well‐known fitness benefits such as those relating to prior residency (Newton & Marquiss, 1982), familiarity with the habitat (Brown, Brown & Brazeal, 2008; Piper, 2011) or breeding with the same partner by maintaining year‐round cohesion (Black, 2001). Here, we propose that associating socially with neighbours during the non‐breeding season also provides individuals with opportunities to encounter and interact with their prior (and potentially future) territorial neighbours. In this way, we highlight that social individuals could also obtain other – so far neglected – benefits in terms of future territory establishment. We thus suggest that forming relationships to offset future territory establishment costs is an example of selection for sociality arising through interdependence (Section IV). We further predict that forming preferential non‐breeding associations within prior breeding neighbourhoods could explain the emergence of complex structured societies (Section V). Our hypothesis thus integrates (and finds support in studies of) non‐breeding social behaviour with carry‐over effects (Section VI).

While we acknowledge that the distinctive shift from seasonal breeding to non‐seasonal sociality is present across most taxonomic groups, it is most widely described in birds (Tobias *et al*., 2016). Hence, we focus this perspective mostly on birds, as these represent the most suitable candidates to test our predictions. Where applicable, we point out research directions and comparisons with other taxa. In doing so, we bridge a major knowledge gap for the non‐breeding season in the avian (Farine, 2025) and, by extension, the vertebrate (Marra *et al*., 2015) literature that, so far, has mostly concentrated on breeding behaviours. Finally, we offer future directions of study (Section VII) and predictions that can be used to test our hypothesis (Section VIII) both in birds and, potentially, other seasonal territorial breeders.

## II. COSTS OF TERRITORIAL BEHAVIOUR SHOULD BE REDUCED AMONG FAMILIAR NEIGHBOURS

We first extend a classical finding in behavioural ecology, the dear‐enemy effect (Temeles, 1994; Fisher, 1954), to incorporate the onset of breeding. Territorial behaviour, whereby an individual defends an area to exclude others for an extended amount of time, is pervasive in the animal kingdom and has received considerable attention (Ord, 2021; Stamps, 1994). Examples come from hunter–gatherers (Peterson, 2009), mammals (Ostfeld, 1990), birds (Brown, 1969), reptiles (Huang *et al*., 2011), amphibians (Bee & Gerhardt, 2002; Mathis *et al*., 1995), fish (Grant, 1993), insects (Baker, 1983) and other invertebrates (Milner *et al*., 2010). By being territorial, individuals can secure preferred access to resources across several niche axes, spanning nesting sites, mates, and/or food. While benefits are usually clear, the costs borne from territoriality are not always straightforward or easily measured (Ord, 2021). These costs can also vary according to the progression of the season, with individuals often incurring the highest costs during the establishment phase when the majority of territorial fights take place (Armstrong, 1991). The costs can greatly impair individual body condition, ultimately affecting short‐ and long‐term survival (Ord, 2021). Therefore, strategies that might remove or mitigate these costs are expected to evolve.

A frequently observed way for individuals to reduce the costs of territoriality is through among‐neighbour familiarity. When neighbouring individuals have a high rate of previous interactions, the resulting familiarity among them can result in lower levels of aggression towards each other. This is largely explained by the fact that existing territory holders (e.g. neighbours) pose a substantially lower risk to a territorial individual as compared to floaters, as the latter may be seeking opportunities to establish their own territory (Temeles, 1994). This phenomenon, first described by Fisher (1954), has been termed the “dear‐enemy effect” (see review in Temeles, 1994) and it has been reported across species in a wide variety of animal taxa (Werba *et al*., 2022; Temeles, 1994). The benefits of having familiar neighbours can be substantial. For example, male red‐winged blackbirds *Agelaius phoeniceus* with familiar neighbours fledge more offspring annually as a consequence of having more females per male territory (Beletsky & Orians, 1989). Similar patterns have also been described for group‐living animals (Christensen & Radford, 2018). For example, groups of green woodhoopoes *Phoeniculus purpureus* have a lower probability of losing a territory when they have familiar neighbours (Ligon & Ligon, 1990). While the dear‐enemy effect among established territory owners has received substantial support (with some notable exceptions where conditions favour higher rates of agonistic interactions, called the “nasty neighbour effect”; Christensen & Radford, 2018), it is worth considering whether it is applicable in other contexts.

One key question is whether the dear‐enemy effect can transcend seasons. In particular, whether it plays a role in buffering the costs involved with territory establishment (in seasonal breeders) within neighbourhoods that contain territory holders from the previous breeding season. If so, then the benefits of having familiar neighbours might extend to the entire yearly cycle through carry‐over effects.

## III. DOES NON‐BREEDING SOCIAL BEHAVIOUR REDUCE THE COSTS OF TERRITORIALITY IN SEASONAL BREEDERS?

The question of whether non‐breeding social behaviour can offer carry‐over benefits in seasonal territorial breeders represents a major research gap. The proximate, short‐term benefits of non‐breeding sociality are now well established [e.g. increased survival (Beauchamp, 2021) or reduced predation risk (Beauchamp, 2004; Sorato *et al*., 2012)]. However, there are substantial opportunities for non‐breeding sociality also to carry over into the following breeding season (Farine, 2025). Here, we suggest three pathways through which non‐breeding social behaviour can reduce territory establishment costs: (*i*) by maintaining familiar relationships from one breeding season to the next; (*ii*) by allowing the communal defence of a shared area during the non‐breeding season; and (*iii*) by increasing non‐breeding survival ultimately to reduce the turnover of neighbours. Each of these pathways would result in the reduction of territory establishment costs and thus select for structured social interactions during the non‐breeding season.

### (1) Sociality can maintain familiarity among past and future neighbours

Animals can often maintain among‐individual social relationships through time, and these entail potential fitness benefits (Silk, 2014). At one extreme, these result in lifelong pair relationships found in some birds, like the laysan albatross *Phoebastria immutabilis* (Rice & Kenyon, 1962), or in the long‐term memory of the calls of relatives in cotton‐top tamarins *Saguinus oedipus* and northern fur‐seals *Callorhinus ursinus* (Matthews & Snowdon, 2011; Insley, 2000). Long‐term relationships can also be maintained with non‐partners and non‐related individuals. For instance, in hooded warblers *Wilsonia citrina*, territory holders can recognize territory neighbours by their song and retain this memory even after the long non‐breeding season (Godard, 1991). Such social relationships can also extend to the colony level, as with slender‐billed gulls *Chroicocephalus genei* that maintain colony membership from one breeding season to the next, even when the colony relocates over large distances (Francesiaz *et al*., 2017). Similarly, in wild zebra finches *Taeniopygia guttata* colonies, individuals can maintain strong social ties both during and after breeding (Brandl *et al*., 2021). Why these relationships are established and maintained through time is still unclear, although the latter two cases appear to have some links to previous reproductive outcomes (colony breeding success in the slender‐billed gulls, and synchronised timing of breeding in the zebra finches).

Here, we highlight that social behaviour during the non‐breeding season can offer a way to establish and/or maintain familiarity and social relationships from one breeding season to the next. Evidence has already shown that during winter (i.e. non‐breeding season), two or more spatially neighbouring cooperative‐breeding units in superb fairywrens, striated thornbills *Acanthiza lineata* or buff‐rumped thornbills *A. reguloides* can coalesce and form higher level social units (Camerlenghi *et al*., 2021; Bell, 1985; Bell & Ford, 1986). Similarly, great tits that spend the non‐breeding season together are also more likely to become spatial neighbours in the following breeding season (Firth & Sheldon, 2016). While social behaviour is likely to have a more prominent effect in resident species, migratory species relocating over large distances during the non‐breeding season can also maintain social relationships (see also Section VII). In migrating long‐tailed tits, families maintained social bonds throughout the southward autumn migration route (Chetverikova *et al*., 2017). Such relationships have also been found among spatial neighbours: European bee‐eaters *Merops apiaster* maintained familiarity with spatial neighbours during migration (Dhanjal‐Adams *et al*., 2018). We thus suggest that dear‐enemy effects could select for non‐breeding sociality if this facilitates the maintenance of breeding neighbourhoods across years.

### (2) Sociality among neighbours can reduce the pool of competitors in the following breeding season

Communal defence of territories during the breeding season, where two or more breeding units share and defend a breeding territory, has been theoretically linked to the evolution of group living and, more generally, sociality (Port, Kappeler & Johnstone, 2011; Rodrigues, Barker & Robinson, 2023). Coalitions – groups of territory owners that collectively defend space against intruders or predators – are favoured by familiarity among territory owners (Getty, 1987). Extending these ideas to the non‐breeding season raises the question of whether previous territory neighbours could communally defend the space on which their territories existed (and will be reformed) to avoid potential competitors from settling there. This would then limit the potential pool of competitors and, thus, offset the costs of having to establish territory boundaries in the presence of potential new territory owners in the future (Mesterton‐Gibbons & Sherratt, 2009). For instance, individuals could actively (*i*) increase agonistic interactions towards unfamiliar individuals (e.g. at food patches), or (*ii*) prevent incoming individuals from joining flocks so that they cannot gain social benefits. We also note that a similar outcome could be obtained through passive processes. For instance, resident individuals could more effectively use up all the resources available in a certain area so that it will appear less profitable to floaters, a process akin to frequency‐dependent resource use (Ogino & Farine, 2024). The ultimate outcome of both active and passive interactions during the non‐breeding season would thus be a decreased social permeability to individuals that did not own a territory in that area previously. If these processes then facilitate the establishment of future breeding territories by reducing the pool of individuals against which they have to compete at the onset of the following breeding season, individuals that help their neighbours will be adaptively favoured.

### (3) Sociality among past and future neighbours can reduce turnover of membership in breeding neighbourhoods

During the non‐breeding season, harsher environmental conditions can lead to higher mortality. For instance, mortality in superb fairywrens peaks in the first part of July, when temperatures are at the lowest and more extreme daily temperature shifts occur (Lv *et al*., 2023). Winter mortality thus contributes disproportionately to individual turnover in seasonal breeders. Factors that can reduce the mortality of neighbours – such as sociality – would thus benefit survivors. For instance, a global analysis showed that flocking behaviour increases annual adult survival, especially in obligate flocking species (Beauchamp, 2021). Similarly, larger breeding units of superb fairywrens had a higher weekly and seasonal survival during winter (Lv *et al*., 2023). Such increased survival rates can be linked to several mechanisms such as a lower predation risk through “safety in numbers” (Guindre‐Parker & Rubenstein, 2020; Lehtonen & Jaatinen, 2016), greater problem‐solving ability (Cantor, Aplin & Farine, 2020), and/or improved thermoregulatory ability (McFarland *et al*., 2015). Recent studies have found evidence for increased cooperation in situations that can bring survival benefits during the non‐breeding season. For example, during the non‐breeding season individual superb fairywrens are more likely to engage in high‐cost antipredator behaviours in response to distress calls by more socially connected individuals (Camerlenghi *et al*., 2023, 2024). Social behaviours and cooperative actions that are directed towards neighbours can reduce their mortality and, in doing so, potentially offset the costs of territory establishment through reduced neighbourhood turnover.

## IV. BUFFERING THE FUTURE COST OF TERRITORY ESTABLISHMENT AS A CASE OF INTERDEPENDENCE

**Fig. 2:**
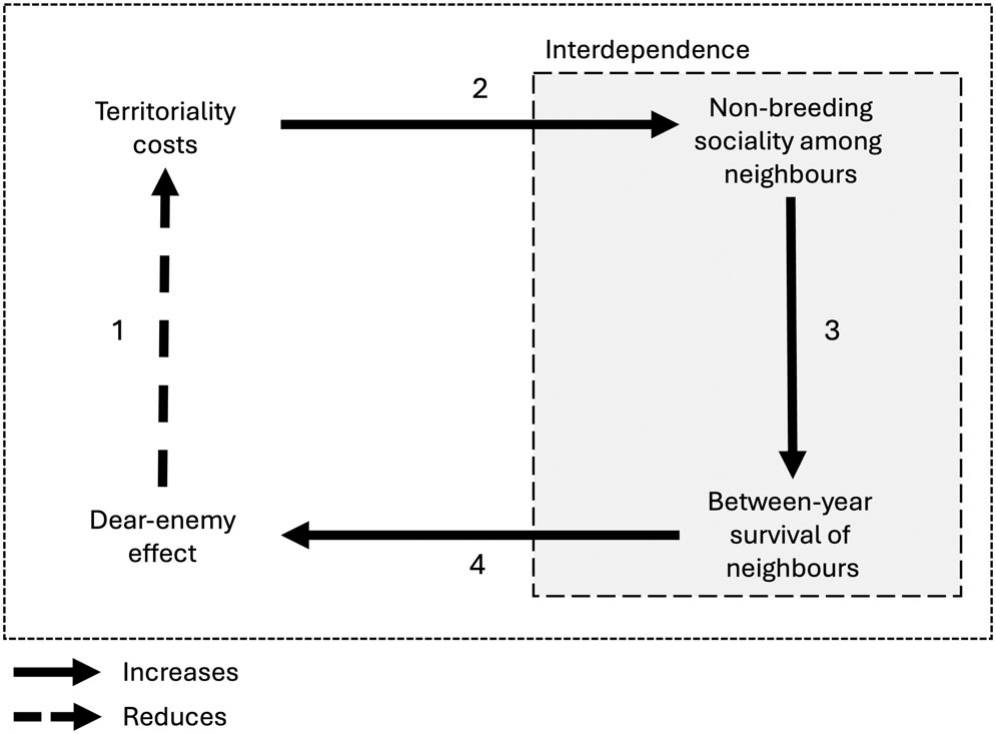
Interdependence can arise when increased survival of previous neighbours during the non‐breeding seasons benefits previous territory owners by mitigating territory re‐establishment costs through dear‐enemy effects. (1) Territoriality costs can be mitigated by reducing aggression towards neighbours that pose relatively lower risk (the dear‐enemy effect). However, territories are also costly to establish. (2) The costs of territory establishment can promote sociality during the non‐breeding season if (3) it increases over‐winter survival (and reduces between‐year turnover among individuals seeking to establish their territory) that then (4) allows prior territory owners to re‐establish their territories with reduced conflict. Behaviours that benefit individuals through the survival of others are known as among‐individual interdependence (shadowed box). Here, interdependence could act to drive associations among previous territory owners through an increase in the stability of the local social environment at the onset of the following breeding season.

While actions that increase the survival of an individual are clearly favoured, it is also possible for individuals to benefit from the survival of others. Obtaining fitness‐enhancing benefits that are gained from the welfare of other individuals is called interdependence (Roberts, 2005). These are well illustrated by the group augmentation hypothesis, whereby individuals that benefit from living in a larger group benefit from the survival or increased reproduction of their group mates (Kokko, Johnstone & Clutton‐Brock, 2001). Here, we extend the interdependence hypothesis to the beneficial carry‐over effects of non‐breeding sociality in seasonally territorial species. Specifically, we suggest that non‐breeding sociality can arise through interdependence if increasing the survival of neighbours (see also Section II) then reduces the future costs of territory establishment (Fig. 2).

## V. BETWEEN‐YEAR DEAR‐ENEMY EFFECTS COULD EXPLAIN THE EMERGENCE OF STRUCTURED SOCIETIES

Social association can provide both immediate (e.g. through avoidance of a predator attack) and longer‐term benefits (e.g. future dear‐enemy effects). While the former can arise through any associations, we expect that the latter should select for social interactions to be preferentially directed towards (some of) these previous neighbours. Thus, the social preferences that arise by mitigating the costs of territoriality (*via* interdependence) are likely to be directed towards a small set of conspecifics, resulting in repeated or stable associations over time. In cooperative‐breeding species, which are commonly territorial (Gaston, 1978) and often year‐round residents, these associations could take place between social groups. In doing so, they could generate patterns of preferential group‐to‐group associations that are known as multilevel societies.

Multilevel societies are hierarchically organized social structures that emerge from preferential group‐to‐group social associations that are stable through time (Camerlenghi *et al*., 2021; Grueter *et al*., 2020, 2021; Papageorgiou *et al*., 2019; Papageorgiou & Farine, 2021). Such social structures are characterised by two or more cohesive levels between the individual and the population (*sensu* Grueter *et al*., 2020). The main social unit of a multilevel society is the “core unit”, a group of individuals that remain stable over time (Grueter *et al*., 2020). Such core units can then coalesce with other core units through molecular fission–fusion dynamics to form one or more higher levels of social organisation (Grueter *et al*., 2020; Grueter, Chapais & Zinner, 2012). A key distinction between multilevel societies and societies with fission–fusion dynamics is that the social decisions to associate (or not) are made by groups and not by individual animals.

Multilevel societies were first described in social primates (Kummer, 1968) and initially thought to be exclusive to large‐brained mammals and humans (Grueter *et al*., 2020). However, similarly structured societies have been long known in birds (Papageorgiou & Farine, 2021), such as bell miners *Manorina melanophrys* (Painter *et al*., 2000), sociable weavers *Philetairus socius* (Ferreira *et al*., 2020) or white‐fronted bee‐eaters *Merops bullockoides* (Hegner, Emlen & Demong, 1982). These are likely to represent examples of multilevel societies, although only two species, the non‐territorial vulturine guineafowl *Acryllium vulturinum* (Papageorgiou *et al*., 2019) and the seasonally territorial superb fairywren (Camerlenghi *et al*., 2021), have been evaluated following the *sensu strictu* definition of multilevel societies (Grueter *et al*., 2020). In the superb fairywren multilevel society, higher level social associations form among two neighbouring breeding groups (Camerlenghi *et al*., 2021). Groups are likely to coalesce into larger non‐breeding social groups to reduce the impact of predation, increase the probability of finding food, and to allow them to access a broader range of resources than those available exclusively in their breeding territory. Here, we propose that the advantages provided by the dear‐enemy effect in reducing future costs of territory establishment among neighbours might be particularly important in explaining the emergence of multilevel societies in seasonal breeding species.

## VI. THE IMPORTANCE OF CARRY‐OVER EFFECTS AND EVIDENCE IN SUPPORT FOR OUR HYPOTHESIS

There is growing awareness that between‐season carry‐over effects have consequences for individuals. Carry‐over effects can take several forms and affect body condition, reproduction, migration, growth rate and physiological performance (Harrison *et al*., 2011). The outcomes can then span from individual to population levels: for instance, demographic changes during the non‐breeding season or during migration can have significant density‐dependent effects during the subsequent breeding season (Ratikainen *et al*., 2008). Even though the importance of between‐seasons carry‐over effects is becoming increasingly recognised, the bias in avian research on the breeding season still persists (Marra *et al*., 2015). This is largely due to the inherent challenges of studying the behaviour of birds when they are not attending a nest (Farine, 2025). There thus remain some large gaps in knowledge about social behaviours – as evidenced from the very recent discovery of multilevel societies in birds (Papageorgiou & Farine, 2021). Here, we highlight a few studies on carry‐over effects that provide preliminary evidence in support of our hypothesis.

Sociality during the non‐breeding season allows much greater benefits than simple dilution of risk. For example, many common good behaviours during the non‐breeding season are directed towards familiar individuals from the previous season. Superb fairywrens perform highly risky distraction displays when exposed to experimental predator models that are directed mainly towards socially connected individuals (Camerlenghi *et al*., 2023). Similar patterns were found in great tits, whereby individuals that maintain long‐term familiarity from the previous breeding season are more likely to engage in communal defence of nests against predators in the following breeding season (Grabowska‐Zhang, Sheldon & Hinde, 2012). Further, maintaining familiar associations with spatial neighbours during the non‐breeding season has also been shown to improve subsequent breeding success. Specifically, brown‐headed cowbird *Molothrus ater* females that maintained more consistent social preferences during autumn laid more eggs during the following spring (Kohn, 2017; Kohn *et al*., 2013). One possible mechanism is that these females need to invest less in competitive interactions at the onset of the breeding season, saving them energy and giving them more time to invest in initiating breeding. Together, these studies suggest that individuals invest in higher cost cooperative behaviours towards individuals that they were familiar with during the previous breeding season, and that the social choices that individuals make during the non‐breeding season can impact their breeding outcomes in the following breeding season.

Such carry‐over effects could also have immediate benefits on individual fitness. For instance, social individuals could attain earlier breeding through reduced territorial conflict (see also Section VIII) with cascading effects on overall seasonal breeding success (Lv *et al*., 2019). Whether that is the case, breeding earlier could allow individuals to avoid overlapping their reproduction with that of their predators. A case in point is offered by the pied currawong *Strepera graculina*–superb fairywren predator–prey relationship (Prawiradilaga, 1996). The pied currawong peak of reproduction coincides with that of superb fairywrens, and the adults mostly provision their nestlings with other species' nestlings, especially those of the superb fairywren. Superb fairywrens that breed earlier will then avoid overlapping their reproduction with that of the currawongs, thus obtaining a higher overall breeding success (Lv *et al*., 2019). Hence, if non‐breeding sociality provides a way to initiate breeding earlier, then social individuals could buffer nest predation rate, with substantial fitness benefits.

In conclusion, while we acknowledge the general lack of studies addressing the carry‐over effects of non‐breeding season social behaviour into the breeding season, we underline that this is likely due to gaps in research effort rather than a lack of biological significance. Hence, in the following section, we provide directions and opportunities to guide future research into the hidden social lives of birds and other vertebrates, which could provide a new, and likely breakthrough, perspective in behavioural ecology.

## VII. FUTURE RESEARCH DIRECTIONS AND TESTING THE HYPOTHESIS

Seasonal territoriality is a widespread strategy among birds, with almost half of bird species maintaining a seasonal or weak territory (Tobias *et al*., 2016). Our hypothesis is thus likely to have relevance for a vast number of bird species. Here, we point out three areas of future directions to explore the link between non‐breeding sociality and the subsequent breeding season.

(1) Population viscosity – a high genetic relatedness among spatially clustered individuals (Wolf & Trillmich, 2008) – is prevalent among sedentary species and can increase interaction rates among kin (Hamilton, 1964). Such a pattern increases the indirect fitness of individuals that cooperate with each other (Axelrod & Hamilton, 1981; Kanwal & Gardner, 2022). Nonetheless, the costs of population viscosity have also been highlighted, both theoretically (Taylor, 1992) and empirically, such as kin competition (West *et al*., 2001) or inbreeding (Harrison *et al*., 2013; Nichols, 2017). The benefits of population viscosity have been also shown in cooperative breeders: for instance, in the southern pied babbler *Turdoides bicolor*, having a kin‐related neighbour resulted in larger territories and kin‐related individuals had shorter territorial interactions (Humphries *et al*., 2021). Similarly, in the group‐living cichlid *Neolamprologus multifasciatus*, related territorial females do not engage in contests with one another, unlike unrelated females (Bose *et al*., 2023). Extending our hypothesis, if individuals associate with their neighbours during the non‐breeding season, it is likely that some of them will also be relatives. Hence, individuals will increase their indirect fitness and future benefits of having a prior neighbour survive (i.e. future interdependence benefits; Roberts, 2005).
(2) In long‐lived species, individuals are more likely to establish long‐term familiar relationships due to the increased probability of repeated interactions (Axelrod & Hamilton, 1981). Several highly social behaviours such as cooperative breeding (Arnold & Owens, 1998; Beauchamp, 2014) or flocking behaviour (Beauchamp, 2021), have been already associated with longer lifespans (Salguero‐Gomez, 2024; Ridley, Yu & Sutherland, 2005). An unexplored avenue for research could examine whether the costs of territoriality decrease proportionally to familiarity, that is whether individuals that maintained familiarity for longer periods experience lower territorial costs. If this is the case, the benefits we highlighted in this review might be stronger for long‐lived species.
(3) While we underlined that the benefits of sociality during the non‐breeding season are relevant for resident species, some migratory species might also be able to maintain familiarity along their migration routes and wintering grounds, as shown in European bee‐eaters (Dhanjal‐Adams *et al*., 2018) and anecdotally in rainbow bee‐eaters *Merops ornatus* (Garnett, 1985). However, contrasting results have been produced, with golden‐crowned sparrows socially associating in stable non‐breeding groups that are subsequently breeding in far apart locations (Block *et al*., 2024). Future research should aim at testing whether and to what extent, social and spatial structuring of territories during the breeding season affects collective migration patterns and how this scales up to population‐level migratory connectivity in non‐breeding areas. In accordance with our hypothesis, we propose some simple predictions that should be testable in field studies (Table 1).
  **Table 1:** Summary of the main predictions of our hypothesis, the underlying rationale and operational guidelines and target species to test them.

Prediction	Rationale	Operational guidelines – data required	Target species
Stronger social associations with previous breeding neighbours during the non‐breeding season will translate to lower rates of agonistic interactions during subsequent territory‐establishment periods.	Both individuals previously shared a territory boundary that can be re‐established.	Social associations during the non‐breeding season. Experimental territorial intrusions during territory establishment (vocal playback, scent and visual territorial intrusions).	Seasonal territorial breeders that form non‐breeding social groups.
Individuals that maintain stronger social associations with previous breeding neighbours during the non‐breeding season will have earlier onset of breeding behaviours and/or greater reproductive investment at the start of the breeding season.	Individuals can re‐direct more time and energy from territorial behaviours to reproductive behaviours.	Social associations during the non‐breeding season. Breeding phenology and/or success.	Seasonal territorial breeders that form non‐breeding social groups.
Stronger social associations with previous breeding neighbours during the non‐breeding season will result in a more stable territory boundary during the next breeding season among these (previous and current) neighbours.	The same territory boundaries can be re‐established with minimal conflict.	Social associations during the non‐breeding season. Year‐to‐year territory boundary stability (e.g. measured as year‐to‐year overlap).	Seasonal territorial breeders that form non‐breeding social groups. Groups of Global Positioning System (GPS)‐tracked long‐distance social migratory species.
Individuals that experience a lower turnover of territory neighbours from one breeding season to the next will have more stable territory boundaries from 1 year to the next.	These individuals can re‐establish their previous boundaries with minimal conflict.	Long‐term data on territory boundaries. Mortality rates across years.	Long‐term/historical studies on seasonal territorial breeders.
Individuals that experience a lower turnover of territory neighbours from one breeding season to the next will have earlier onset of breeding behaviours and/or greater reproductive investment at the start of the breeding season.	Individuals can re‐direct more time and energy from territorial behaviours to reproductive behaviours.	Long‐term data on territory boundaries. Breeding phenology and/or success.	Long‐term/historical studies on seasonal territorial breeders.
There should be fewer associations during the non‐breeding season with non‐local individuals (i.e. fewer intrusions) at the core of the prior joint territory area than at the periphery of this area (or beyond this area).	Individuals would benefit from jointly preventing non‐local individuals from settling and potentially competing for territories in the following breeding season.	Social association data during the non‐breeding season. Territory boundaries of the previous breeding season.	Seasonal territorial breeders that form non‐breeding social groups. Seasonal territorial breeders that communally defend a non‐breeding area.
Individuals that maintained familiarity for longer periods through both breeding and non‐breeding seasons should experience lower territorial aggression.	Costs of territoriality should decrease proportionally to familiarity.	Long‐term social associations data. Experimental territorial intrusions at territory establishment (vocal playback, scent and visual territorial intrusions).	Long‐lived seasonal territorial breeders.

## IX. CONCLUSIONS

(1) One of the major open questions in the study of territoriality is how individuals buffer the costs associated with establishing and maintaining their territory. Two prevalent strategies to cope with these costs are seasonal territoriality and the dear‐enemy effect. While almost always considered independently, these two strategies are likely to be interacting through social behaviour during the non‐breeding season.
(2) Our hypothesis proposes that associating preferentially with previous territory neighbours could offer a way to buffer the future costs of territory establishment. If this is the case, then sociality during the non‐breeding season could evolve *via* interdependence, whereby individuals benefit from the increased proportion of neighbours that are maintained to the following breeding season. Our perspective thus opens a promising research avenue on the interaction between seasonal territoriality, the dear‐enemy effect and social behaviour in seasonal territorial breeders. This could offer a much deeper understanding on the neglected non‐breeding season and the carry‐over effects onto and from the breeding season.
(3) Explicitly addressing the interconnections between seasons, thus adopting a full‐annual‐cycle approach, will provide significant insights into why individuals establish and maintain social connections with others, and what the consequences of these associations are. Such deeper understanding will provide further insight into the evolution of complex and structured social systems in both birds and other seasonal territorial breeders, providing key elements to understand their drivers and consequences for individuals.
